# The impact of cultural healthcare practices on Children’s health in the United Arab Emirates: a qualitative study of traditional remedies and implications

**DOI:** 10.3389/fpubh.2023.1266742

**Published:** 2023-10-06

**Authors:** Nabeel Al-Yateem, Amal Muneer Abobakr Lajam, Mariam Mohamad Gouda Othman, Maryam Ahmed Ali Ahmed, Shayma Ibrahim, Aram Halimi, Fatma Refaat Ahmad, Muhammad Arsyad Subu, Jacqueline Maria Dias, Syed Azizur Rahman, Ahmad Rajeh Saifan, Heba Hijazi

**Affiliations:** ^1^Department of Nursing, College of Health Sciences, University of Sharjah, Sharjah, United Arab Emirates; ^2^School of Nursing, Paramedicine and Healthcare Sciences, Faculty of Science and Health Charles Sturt University, Bathurst, NSW, Australia; ^3^Student Research Committee, School of Public Health and Safety, Shahid Beheshti University of Medical Sciences, Tehran, Iran; ^4^Department of Epidemiology, School of Public Health and Safety, Shahid Beheshti University of Medical Sciences, Tehran, Iran; ^5^Department of Critical Care and Emergency Nursing, Faculty of Nursing, Alexandria University, Alexandria, Egypt; ^6^Faculty of Nursing and Midwifery, Binawan University, Jakarta, Indonesia; ^7^Department of Health Service Administration, College of Health Sciences, University of Sharjah, Sharjah, United Arab Emirates; ^8^Applied Science Private University, Amman, Jordan; ^9^Department of Health Management and Policy, Faculty of Medicine, Jordan University of Science and Technology, Irbid, Jordan

**Keywords:** cultural healthcare practices, Children’s health, United Arab Emirates (UAE), traditional remedies, home treatments, qualitative study, cultural identity

## Abstract

**Aim:**

This qualitative study investigates the impact of cultural practices on children’s health in the United Arab Emirates (UAE) by examining the use of traditional remedies and home treatments by mothers.

**Methods:**

Twenty-five participants, all mothers who had employed traditional treatments or home remedies for their children during periods of illness, were included in the study. The participants represented a diverse range of educational backgrounds, from school diploma holders to university degree graduates, with ages spanning from 20 to 50 years. Hailing from different Arabic countries and cultural subgroups, the majority of participating mothers were from the UAE.

**Results:**

Through in-depth interviews, three major themes emerged from the participants’ experiences. Firstly, a strong connection between culture, religion, and healthcare practices was evident. Many mothers opted for cultural remedies as their first line of defense against illnesses due to the practices’ strong foundations in their cultural heritage. Herbal remedies, Quranic healing, and other traditional methods were perceived to be both effective and spiritually comforting, reinforcing participants’ sense of cultural identity. Secondly, participants highlighted unintended consequences of relying solely on traditional treatments. Some instances were reported where the use of ineffective remedies resulted in delays in seeking appropriate medical care for their children, potentially compromising their health. Additionally, certain misconceptions regarding the safety and efficacy of traditional remedies were identified, emphasizing the need for evidence-based healthcare education.

**Conclusion:**

This qualitative study sheds light on the intricate interplay between culture, traditional remedies, and children’s health in the UAE. The incorporation of diverse participants from various Arabic countries and cultural subgroups enriches the study’s applicability to broader Arabic cultures. By recognizing the significance of cultural healthcare practices and striking a balance with evidence-based care, healthcare providers can create a more inclusive and effective healthcare environment for children in the UAE. Future research should explore diverse samples and develop targeted interventions to further advance cultural awareness and understanding in healthcare practices.

## Introduction

Child health is an intricate and multidimensional phenomenon that is shaped by biological factors and profoundly influenced by socio-cultural contexts. Culture, in the context of this study, refers to the shared practices, beliefs, norms, and values that are passed down through generations and are influenced by factors like religion, ethnicity, and societal structures. In Arabic and Muslim cultures, cultural practices and beliefs play a pivotal role in children’s health outcomes. These practices are steeped in deep traditions and social norms and dictate various aspects of a child’s life, including nutrition, access to healthcare, and the management of illnesses. The impacts of these practices on child health can be both positive and negative, and rigorous investigation is needed to tailor effective interventions. This study explored the intersection between cultural practices and child health in the Arabic and Muslim context of the United Arab Emirates (UAE).

## Background

The UAE is situated in WHO’s Eastern Mediterranean region and embraces a framework shaped by its unique historical, religious, and cultural experiences. This framework is deeply rooted in the principles of Islam and specific Arabic traditions that have been nurtured and evolved over time. Social and economic progress in the UAE over the last half-century has been guided by these cultural and religious beliefs, which have provided a unique developmental context. Formerly a nomadic society, the UAE has seen a rapid transition to structures and systems similar to those in contemporary technologically advanced countries. In the area of healthcare, the transformation has been dramatic and swift, with the introduction of extensive medical amenities and services. Despite these advances, the UAE has struggled to foster sufficient local human resources development, particularly in the healthcare sector. The rapid expansion of hospital facilities and the ambition to provide a broad spectrum of quality healthcare services have exacerbated the lack of adequate local healthcare workers, especially nurses (1, 2). To address the shortage of healthcare workers, the UAE has relied on recruiting expatriate nurses from various ethnic and cultural backgrounds. However, this international workforce is often unfamiliar with the Arabic language, Islamic principles, and local cultures and values. This cultural knowledge gap may pose significant challenges and have detrimental consequences for both patients and healthcare providers. For example, patients receiving culturally incompetent care may experience dissatisfaction and potentially negative outcomes, which affect both patients and their families. The importance of cultural competence is underscored by evidence suggesting patient outcomes are better when healthcare is delivered with cultural sensitivity and understanding (3–8). However, the expatriate nursing workforce is the backbone of the healthcare sector and plays a crucial role in the UAE. Therefore, addressing their lack of cultural knowledge and enhancing their understanding of local customs, values, and the Islamic way of life is important to improve patient satisfaction and help avoid negative outcomes of some cultural practices.

### Cultural practices and child health

Being a traditional society, the UAE’s understanding and treatment of illness often extends beyond the mere confines of the biomedical model. They encompass treatment options that include not just medicines but also rituals, herbal remedies, and community ceremonies. Coupled with challenges in the healthcare service, where clients must navigate a highly modernized system, staffed with linguistically and culturally different healthcare professionals, these factors can shape the health beliefs and behaviors of clients. Such beliefs and behaviors can be influenced by the perceived benefits of adhering to cultural norms and the perceived barriers to adopting modern healthcare practices (9).

In the literature, many cultural practices have been reported to affect different aspects of children’s lives; for example, nutritional practices are deeply embedded in culture and have a significant impact on child health. Studies conducted in diverse settings found that cultural beliefs and practices influenced decisions about infant feeding and weaning, including the types of foods introduced (10–13). However, such practices often clash with scientifically recommended approaches (e.g., exclusive breastfeeding for the first 6 months). The effects of cultural norms on child nutrition extend beyond infancy. A previous study found that food-related cultural norms influenced food selection for primary school children, leading to unbalanced diets (14). Cultural influences on feeding practices that impacted child nutrition were also reported among immigrant communities living in high-income countries (e.g., Australia), which may lead to child health disparities (e.g., high obesity rates) (15).

Cultural practices also play a significant role in parental health-seeking behavior and treatment choices. In some African cultural subgroups, mothers’ social strategies and cultural practices have been reported to result in inconsistent health-seeking behavior and impede long-term solutions for their children’s illnesses (16). This scenario becomes more complex where local perceptions and reliance on traditional healers overshadow conventional healthcare practices (17). Studies conducted on the Kenyan coast and among Syrian refugee parents in San Francisco highlighted that traditional healing systems often coexisted with biomedical systems, creating a dichotomy that sometimes led to sub-optimal health-seeking behavior (18). Several studies focused on specific areas of child health (e.g., burns, skincare, traditional medicine use) and cultural and spiritual beliefs underlined the potential risks posed by some cultural practices, and highlighted the role of cultural perceptions in shaping health outcomes. For example, Abraar Karan et al. (19) and Skinner and Peat (20) found that increased public education could reduce burn injuries and their associated complications (19, 20). Another study focused on skincare recommended more research on practices such as early bathing and the use of certain emollients in some cultural groups, which may disrupt the skin barrier and lead to preventable conditions (e.g., atopic dermatitis) (21). Cultural practices were also found to significantly impact children’s sleep patterns (e.g., sleep duration and disturbance) (22), which underscored the need for further research in this area (22, 23).

Traditional medicine continues to play a significant role in child healthcare in many Arabic and Muslim contexts. Ngere et al. (24) found an increased demand for traditional medicine in Western Kenya, with caregivers often preferring traditional approaches to treat certain childhood diseases. That study highlighted the need to improve the safety and quality standards of traditional medicine products (24). The complex interplay between cultural practices and child health requires a nuanced understanding and targeted interventions. Although existing research provides substantial evidence for the importance of understanding the local cultural practices and its effect on health, this area has not yet been explored in the UAE and surrounding region. As research in regions such as the UAE continues to grow, it is vital to remain conscious of cultural dynamics to enhance population health and well-being, particularly among children, especially as the Islamic and Arabic population in the UAE remains adherent to their cultural practices.

## Study aim

This study aimed to investigate parents’ perceptions of health-related cultural practices adopted by families, particularly during instances of child illness. We also explored the potential effects of these cultural practices on children’s health and the way these practices may have influenced parents’ decisions to seek medical health services.

## Methods

### Study design

An exploratory qualitative research approach that borrowed from phenomenology principles was deemed most suitable for this study as we intended to explore participants’ experiences. Phenomenology was used to guide the sample selection, data collection, and data analysis processes. Parents and family members of children who had experience of using cultural remedies while caring for their child in general and during illness were purposely chosen to participate. Participants’ narratives and experiences were carefully examined through individual interviews, which then underwent phenomenological data analysis as proposed by Van Manen (25). Van Manen’s methodology aims to achieve a subjective description of experiences but also calls for understanding these experiences (25). This philosophical stance allowed us to explore how traditional practices may have affected children’s health and parents’ medical help seeking.

### Sample and settings

This study included parents of children attending maternal and child healthcare centers in the UAE who were receiving care for themselves or their children. Visitors to these centers are typically parents—mainly mothers—seeking treatment for themselves or their children, vaccination or child development consultations, and wellness checks. Purposive sampling was used to select participants who had experience of using traditional treatment methods with their children. The number of participants was determined by the principle of data saturation, whereby interviewing continued until no new data emerged. Parents from multiple cultural backgrounds living in the UAE were selected, particularly those who spoke Arabic. The interviews took place in rooms in the healthcare centers that had been booked and prepared for this purpose. Alternative locations were arranged for participants who preferred to meet outside these centers.

### Data collection methods

Data were collected in qualitative semi-structured interviews. An interview guide was prepared in advance that contained the areas that need to be covered during the interviews. The questions in the interview guide offered general framework for the interview; however, the researchers were open to including other questions to explore areas mentioned by participants. The questions were open-ended to gather maximum information from participants. The interviews were recorded and then transcribed for data analysis. Examples of the interview questions are as follows. “Can you tell me about a time when you used a traditional treatment or home remedy for your child’s illness?” “What prompted you to use it?” “Could you describe a situation where the home remedy or traditional treatment seemed to work effectively?” “Can you recall an instance where the remedy or treatment may have had a negative impact on your child’s health?” “What happened then?”

### Data analysis

**Figure 1:**
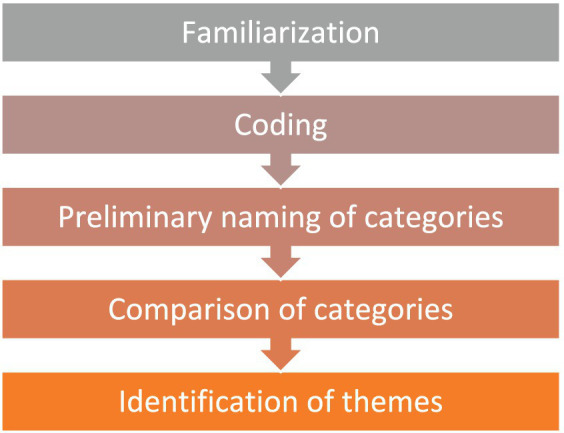
Van Manen’s data analysis process.

The phenomenological accounts of participants’ experiences were analyzed using the framework proposed by Van Manen in 1990 (Figure 1). This approach offered an explicit and clear method for data analysis. Data coding was performed by two independent researchers with expertise in qualitative research. After the initial round of coding, both researchers met to discuss and reconcile any differences in their coded data, ensuring consistency and reliability in the coding process. Cross-checking was done by having a third senior researcher review a subset of the data to confirm the coded themes and offer feedback. For the purpose of this study, we employed the NVivo software to facilitate the organization and analysis of the data.

### Ethical considerations

Ethical approval to conduct this study was obtained from the Research Ethics Committee of the University of Sharjah. Written consent was obtained from all participants, and participants were informed that the information they disclosed in their interview would be kept confidential. To maintain participants’ confidentiality and anonymity, participants were anonymized (using numbers) for all documentation, with these numbers used throughout the study. The coding page and interview records were kept in a locked cabinet that was only accessible to the research team.

## Findings

### Study participants

This study included 25 participants (i.e., 25 interviews). All participants were mothers who had used traditional treatments or home remedies for their children when they were ill. Participants had differing educational backgrounds (e.g., from school diploma to university degree), and their ages ranged from 20 to 50 years. Participating mothers belonged to different Arabic countries and cultural subgroups, with a majority from the UAE. In our quest to delve deep into the lived experiences of our participants, we conducted comprehensive interviews with each of them. The interviews ranged from 30 to 45 min, with an average duration of 35 min per interview. This extensive engagement allowed us to gather in-depth insights, capturing the nuances and intricacies of their experiences, ensuring the richness of our data.

### Study themes

**Figure 2:**
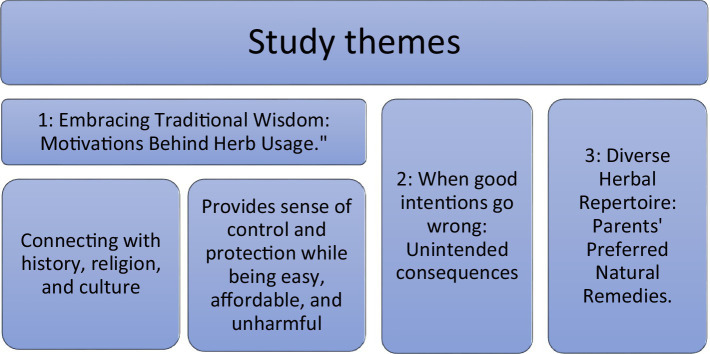
Study themes.

Three major themes that represented the experiences of study participants in using cultural healthcare practices with their children were identified (Figure 2).

#### Theme 1: embracing traditional wisdom: motivations behind herb usage

##### Connecting with religion and culture

Many Arab participants reported that they often turned to cultural health practices as their first line of defense against illness, rather than relying on scientific medical treatments. This was because these practices and traditions had a strong foundation in their cultural heritage, and were seen as a way to connect with their culture and heritage and maintain the health and well-being of the family. Some of these practices included the use of herbal remedies and the consumption of certain foods and drinks that were believed to have medicinal properties.

*“…for me, if I see my child’s situation as mild, I will take care of him at home and will use natural remedies.” (Egyptian mother).*

*“Yes, I always use remedies I learned from my family and myself used to take; if it did not work, then I will take them to [a] doctor.” (Emirati mother).*

A few participants shared that they had tried some religious and spiritual practices. For example, the Quran was believed to have healing properties and was used as a means of reducing illness. This practice is often referred to as Quranic healing, and involves reciting specific verses from the Quran to cure illnesses. It is thought to create a soothing effect for the patient’s mind and body, which helps in the healing process.

*“As a mom, I turn to the Quran when my child is unwell. Its verses, for me, have a healing touch. They comfort us, help my child feel better, and in our own way, bring us closer to our faith.” (Chadian mother).*

Some mothers said that these practices and remedies reminded them of their families, especially their mothers, and how they treated and cared for them when they were children. By using these remedies, they felt connected with their heritage and like their own parents.

*“Using these home remedies, I’m often reminded of my own mom. I can almost see her caring hands treating us. It’s a beautiful feeling of similarity, of walking in her footsteps. She did great things for us, and I hope to do the same for my children.” (Emirati mother).*

##### Provides sense of control and protection while being easy, affordable, and unharmful

Traditional approaches were used for a variety of reasons. It was believed that natural remedies were less invasive and had fewer side effects, and that they provided a sense of control and empowerment for parents. Home remedies were also seen as a cost-effective option for treating minor illnesses and injuries.

*“Being a mom can sometimes feel like a juggling cat. But with home remedies, I feel like I’ve got more control. It’s my way of doing things, my way of looking after my little ones.” (Emirati mother).*

*“Natural remedies, in my experience, do not have as many side effects. Plus, they are easier on the wallet. Who would not want a solution that’s simpler, safer, and saves us a bit of money too?” (Jordanian mother).*

Unlike modern medicine that only treats the illness or the problem, traditional and religious remedies were perceived as protecting the person and preventing illnesses or even the “people devils.” For example, some participants mentioned that the evil eye could cause sickness in their children. This was defined as a curse cast by a jealous person, which could cause harm or sickness. Some parents recited prayers and Quranic verses to seek protection from Allah or consulted with religious or traditional healers who specialized in removing the effects of the evil eye.

*“Modern medicine helps with sickness, sure. But our old ways, like using prayers and verses from the Quran, they protect us from more, even from things like the evil eye.” (Egyptian mother).*

*“When my child is unwell, it’s not just about treating the illness. I recite prayers, ask Allah for protection, and sometimes, I seek help from healers. We believe these steps help keep harm at bay.” (Female, mother).*

One participant mentioned that reading Quranic verses over water could infuse the water with healing properties and make it more beneficial for drinking and other purposes. The water could then be used in various ways, including drinking, bathing, or applying it to affected areas of the body. Participants believed that this could only be useful, and would never cause side effects as modern medicines often did.

*“I’ve often found comfort in the power of the Quran. In fact, I sometimes read its verses over water, believing it to imbue the water with healing properties. We use it for drinking, bathing, even applying it where it hurts. Unlike modern medicine, this way carries no fear of side effects—just pure, gentle relief.” (Emirati mother).*

Sometimes these remedies were the only option available for the family, as some participants did not have access to reliable health information or were not aware of the appropriate medical interventions for their child’s condition. Therefore, they relied on home remedies as a first line of action. In addition, some parents preferred a natural approach to healthcare and felt more comfortable using home remedies instead of medication.

*“As a mom, sometimes home remedies are my first and only option. Not everyone has easy access to doctors or knows what steps to take when their child falls sick. So, we lean on what we know—these natural, homegrown cures. They’re a comfort, a reliable fallback when the world of medicine feels too far away or too overwhelming.” (Emirate mother).*

One factor that pushed some participants to try home and traditional remedies despite their suspicions that this approach may not work was a previous negative experience with a healthcare professional or health facility, such as feeling rushed, not being listened to, receiving a misdiagnosis, or experiencing communication barriers. These experiences led to a lack of trust in future interactions.

*“I’ve had times where doctors seemed too busy to really listen or they got something wrong. These experiences made me cautious. So, even if I’m unsure about home remedies, I still give them a try. They give me some control when I feel like the medical world has let me down.” (Emirati mother).*

The fact that the majority of healthcare professionals were from various countries around the world and often lacked fluency in the local language created a barrier. To navigate this, participants reported that they first tried to avoid visiting these professionals and instead turned to local traditional remedies. If these remedies proved effective, it was ideal; otherwise, they sought professional medical treatment.

*“It can be tough with doctors from different places; we do not always understand each other. That’s why I try our old home treatments first. If they work, perfect! If not, then we’ll just have to see the doctor and try our best to understand each other.” (Emirate mother).*

Some participants believed that herbal medicine was superior to doctors’ prescribed medicines. The latter were perceived to weaken the body and cause side effects, whereas traditional and herbal medicines were believed to strengthen the body and have no side effects at all. Interestingly, there was a juxtaposition in the practices, as exemplified by one participant who, while favoring natural remedies like lemon, honey, and turmeric, also turned to biomedical solutions such as applying ibuprofen powder topically for pain and inflammation. This demonstrates a blending of both traditional and biomedical approaches in their health practices.

*“As a mom, I’ve seen that hospital medicines seem to make illnesses come back more often and get worse each season. My child gets sick quickly because his natural immunity is not strong. It feels like these medicines weaken the body more than they help, even for grown-ups. I prefer natural things like lemon, honey, turmeric, coriander, chamomile, and sage. They do not have any side effects like those medicines which can hurt the kidneys over time. Even for pain, I use ibuprofen powder mixed with water and put it right where it hurts or is swollen.” (Yemeni mother).*

#### Theme 2: when good intentions go wrong: unintended consequences

Some participants’ narratives indicated that using ineffective remedies could fail to improve a child’s condition or may even worsen it. This resulted in delays in seeking proper medical care and potentially compromised the child’s health. However, many participants felt that cultural practices were a part of their identity, and must be continued regardless of their efficacy.

*“Sometimes, our cultural remedies might not work as we’d hope and might even delay us from getting the right medical help. But these practices, they are a part of who we are.” (Emirati mother).*

Many participants tried remedies that they had learned from their parents, family members, social media, or friends, and later came to regret it. They realized that while these home remedies may have some benefits for certain individuals, they were not always effective or safe for every condition, particularly for children. Having learned the hard way, they indicated they understood the importance of consulting a healthcare professional before trying any home remedies or alternative treatments for children, especially if the child had an underlying medical condition or was receiving other medications.

*“I remember my cousin’s kid had an ear infection. She tried treating it by putting olive oil in the ears of the kid, but it only made it worse. And a friend of mine tried using onion water for her boy’s flu, but he ended up with sinusitis. I think the water was too hot for him. So, you see, these home remedies, if we do not use them right, can cause more harm than good.” (Emirati mother).*

*“I learned from a family member that if you have an earache, you can just put a piece of onion in your ear for a bit and it’ll get better. I tried this with my daughter, Maryam, when she had an ear infection. But it did not help; it actually got worse, and we ended up needing to go to the hospital.” (Yemeni mother).*

One misconception that was observed related to the frequent use of honey when children fell ill with various conditions, such as tonsillitis, cough, and even for general strengthening and detoxifying of the liver and body. Honey was often administered mixed with other herbs and citrus fruits, such as lemon and orange. However, honey is not recommended for children under the age of 1 year, as it may lead to serious illnesses such as infant botulism, or more minor issues such as nutritional imbalances and tooth decay because of its high sugar content. There is also a risk for choking if the child is very young.

*“I gave my children honey when they were 40 days old. It is good for jaundice and helps cleanse the liver.” (Yemeni mother).*

*“As a mom, I’ve heard from others that honey is like a magic cure! Mixed with lemon, it’s used for coughs, colds, and even asthma. Some say it helps with a sore throat and keeps the liver healthy.” (Emirati Mother).*

Another noticeable practice was the use of Bukhoor, which is a Middle Eastern incense, to protect children and keep the home smelling fresh and nice. This remedy was also sometimes used when the child was ill, especially with a respiratory illness, to decrease cough and clear secretions in the chest. However, burned scents (e.g., Bukhoor), can be harmful if inhaled excessively, especially for children with respiratory issues. The smoke and particles may exacerbate respiratory problems and allergies, and prolonged heavy use may even increase the risk for asthma and cancer. It is important to note that asthma is among the top illnesses among children in the UAE.

*“I use Bukhoor to keep my kids safe from bad vibes, especially when they are crying a lot or we are having people over. It also makes our place smell nice. Plus, when they are sick, it seems to help with their coughs and chesty colds. That Arabic gum Bukhoor works especially well.” (Emirati mother).*

#### Theme 3: the ultimate guide to natural remedies for your little ones: from honey and ginger to onion!

Participants frequently mentioned the use of herbs and traditional remedies in their responses. A wide range of spices and herbs, including turmeric, cloves, cumin, anise, za’atar, mint, ginger, chamomile, and *Nigella sativa*, were cited as beneficial, especially during periods of their children’s sickness. The specific methods of use and combinations varied among participants. For instance, one mother shared her recipe to boost immunity:

*“During corona I used to give my kids the herbal remedies to strengthen their immunity and protect them from illness. I gave them honey with orange and lemon and honey with the black seeds [*Nigella sativa*].”*

Another participant described how turmeric was useful for gas and abdominal aches. Turmeric is traditionally used for disorders of the skin, upper respiratory tract, joints, and digestive system.

*“I always use fresh, yellow Indian ginger for my child. You just need to cut it into small pieces and have them use it. It helps their body quickly and I often find their illness is gone by the next day.” (Emirati mother).*

Another participant used turmeric as a cream to relieve pain and decrease swelling. She said that the strong properties of turmeric assisted to reduce inflammation and guard against potential health problems.

*“I remember a time when I was young; my hand was injured and swollen. My granny used turmeric mixed with dates and applied it to my hand. By the next day, the pain and swelling were gone.” (Emirati mother).*

One participant shared a recipe that she used for her children when they had the flu or a fever.

*“The last remedy I had for pain and fever in general is a mixture made of olive oil, coarse salt, ground cinnamon, ground cloves, Vicks, and Vaseline. I cook them over low heat, then cool them down and put them in containers. I use them during fever and flu cases to rub on the body.” (Emirati mother).*

Another participant shared a recipe for combating tooth decay in her children.

*“Clove paste mixed with water is considered a killer of bacteria and is used to treat tooth pain and decay.” (Emirati mother).*

Other participants mentioned using cumin for abdominal gas and vomiting, as it served as an antioxidant in the body and helped cleanse the stomach of toxins.

*“Some herbs are more beneficial for stomach gasses than the medicines that doctors prescribe. We even use herbs as an alternative to vitamin D. For gasses, we use cumin and anise, which are more helpful. They are also beneficial for those who vomit a lot.” (Emirati mother).*

*“Once my son had colic, so I gave him boiled cumin. Afterward, he vomited, but it was all toxins and dirt being cleared out. Thankfully, he felt better afterward.” (Yemeni mother).*

Anise was mentioned multiple times as an herb used to treat upset stomach, intestinal gas, and runny noses.

*“My mom used to give us anise and dates with water. I give my children the same mixture, and it helps them with colic and gas. It’s considered a prophetic medicine before being a folk remedy.” (Moroccan mother).*

One participant shared how giving her child za’atar, he became more focused and energetic.

*“Ever since my child started having za’atar as part of his daily meals, I’ve seen a boost in his energy and focus. He seems healthier too! It seems to have improved his immune system, blood circulation, and even cleared his airways.”*

Many participants shared that onion could be used as a home remedy for fever and inflammation. They believed that onion had antibacterial and fever-reducing properties. There were different methods of application, one of which was the onion poultice technique. This involved placing a slice of onion on the bottom of the feet, covering the feet with socks, and leaving them on overnight. It was believed that this helped draw out toxins and reduce fever.

*“When they have a fever, I use onion at the bottom of the foot; it absorbs the heat.” (Emirati mother).*

In addition, many mothers used water infused with other ingredients such as dates and sugar. This practice was based on the belief that dates have numerous health benefits and can provide essential nutrients for growing children. Date water was made by soaking dates in water overnight or for a few hours, then blending the mixture until smooth. This traditional practice was a testament to the resourcefulness of mothers in using natural remedies to support the health and well-being of their children.

*“I give my children everything from their first 40 days. This includes herbs, cooked food, and dates. I give them water with dates or water with sugar.” (Emirati mother).*

Finally, participants reported they used gum Arabic to treat respiratory problems, such as asthma and bronchitis, as well as digestive issues and skin conditions. Many participants said that they used Bukhoor to treat cough and clear secretions.

*“I also use gum Arabic in treating abscesses or ulcers. I put a small amount on the area.” (Egyptian mother).*

## Discussion

Research on the influence of culture on children’s health in the UAE is limited. This study fills a crucial knowledge gap and offers insights into potential areas for health promotion and disease prevention among children in the UAE. By understanding cultural practices, healthcare providers can enhance their cultural competency, which will lead to more culturally sensitive strategies and ultimately, improved health outcomes for children. The importance of this study is further emphasized by the fact that most healthcare professionals working in the UAE are from overseas, and therefore come from different cultural backgrounds. As such, they are unfamiliar with the local culture and need appropriate education in this area. Moreover, the UAE healthcare workforce is transient, which results in a continuous knowledge gap regarding local cultural practices and their intersection with healthcare services.

Using a qualitative interview method, we gained profound insights into individuals’ experiences and perspectives about traditional home remedies in the UAE context, which has enriched our understanding of the effect of Arabic cultural practices on child health. The diversity of participants, which included individuals from the UAE and many other Arabic countries, increased the applicability of our findings to broader Arabic cultures. Participants in our study exhibited strong enthusiasm about sharing their cultural practices and beliefs, which they considered essential in preserving their cultural heritage and promoting their children’s health. Implementing these practices also appeared to serve as a therapeutic method for their children during illness and also for parents, as it fostered a sense of connection to their past and memories of the places and people they cherished. A critical finding of this study was parents’ strong reliance on cultural practices and beliefs in managing their children’s health. Many parents preferred traditional remedies, such as honey and teas, for common childhood illnesses. However, evidence suggests that such practices may contribute to nutritional deficiencies in children. This highlights the need for healthcare providers to promote evidence-based nutrition practices. Our findings also emphasized a major concern regarding potential delays in seeking appropriate medical care because of reliance on traditional, spiritual, and religious treatments. Previous research supports this concern, given the high risk for preventable deaths among children whose parents relied on traditional and faith healing (Journal of Religion and Health).

An interesting finding in this study was that participants’ cultural and religious connections to traditional health practices, rather than reliance on scientific medicine, could be attributable to the fact that these practices offered comfort and a sense of control, were cost-effective, and had fewer side effects compared with modern medicines. They also offered a sense of protection from both illness and harmful spiritual influences. Interestingly, this study also highlighted challenges parents faced with the UAE healthcare system, including past negative experiences and communication barriers, which appeared to reinforce their reliance on home remedies. Many studies have reported issues related to communication barriers and lack of culturally competent care in healthcare systems, and highlighted the potential impact on the quality of services provided and the experiences of service users. Nursing care that lacks cultural competence can result in patient dissatisfaction and detrimentally affect outcomes for both patients and their families. This contrasts with the positive results that can be achieved with culturally competent care (3–8, 26, 27). While this study does highlight the urgency for healthcare providers to promote evidence-based practices, it is essential to recognize that simply acknowledging the existence of traditional remedies is not sufficient for a provider to be considered culturally sensitive. Respecting and understanding these practices go beyond mere recognition; it requires a deeper comprehension and validation of the cultural and emotional roots tied to these remedies. Instead of approaching these traditions with a primary intent to educate or correct, healthcare providers must first establish trust and rapport with patients and families. This is particularly vital in the UAE, given the diverse backgrounds of both the healthcare workforce and the population. Training healthcare providers in effective communication skills, tailored to the specific cultural nuances of the region, could foster this essential trust. This would not only ensure the preservation of cultural heritage but also facilitate better understanding and collaboration between patients and providers. Through such meaningful interactions, providers can both respect traditional practices and gently guide families toward evidence-based healthcare decisions, mitigating potential risks without dismissing deeply held beliefs.

This study shed light on the potential risks of using home remedies. Although these traditional practices are deeply ingrained in the cultural identity, they may not always be effective or safe. Participants reported instances where these remedies had worsened their child’s condition, and examples of using remedies against which scientific evidence exists. This emphasized the urgent need to address this issue at high levels of the healthcare system. The “encyclopedia” of remedies offered by participants in this study reflected the popularity and diversity of the home remedies used. Parents frequently reported using a variety of herbs and spices as remedies, with recipes often passed down through generations and reflecting a collective cultural memory of healing practices. Strategies to address issues associated with use of such remedies should focus on raising awareness about the risks of relying solely on traditional spiritual and religious treatments. Education, community outreach programs, media campaigns, and potential regulation of certain practices are promising strategies for addressing this issue. Furthermore, our study revealed some misconceptions among parents who believed that ineffective home remedies would not cause harm to their children’s health. However, some plants that are considered beneficial can cause adverse reactions in children, particularly when age and weight are not carefully considered. For example, a 2018 study published in the *Journal of Clinical and Experimental Hepatology* linked certain herbal teas and supplements to liver damage in children, underscoring the need for parental awareness about potential risks.

## Conclusion

This study underscores the critical role that cultural practices play in the management of children’s health in the UAE and other Arabic cultures. The strong reliance on traditional remedies and spiritual treatments is deeply rooted in cultural and emotional ties, chosen for their perceived comfort, control, cost-effectiveness, and few side effects. However, there are tangible risks associated with such practices, including nutritional deficiencies, delays in seeking appropriate medical care, and potential adverse reactions.

While it’s vital to enhance the cultural competency of healthcare providers, especially those from diverse cultural backgrounds, it’s equally important to approach these traditional practices with genuine respect and understanding. Efforts should extend beyond merely recognizing these practices; they should involve deep comprehension and validation of the cultural and emotional significance they hold for families. Enhancing trust between healthcare providers and families can pave the way for more collaborative and informed healthcare decisions.

Furthermore, there is a need to carefully educate parents about potential negative effects of some traditional treatments if used for their children without proper guidance, ensuring this education is done without dismissing the cultural significance of these practices. This balance can be achieved through culturally sensitive education, community outreach programs, and potential regulation of certain practices. Finally, further research should extend the scope to diverse samples and explore the efficacy of targeted interventions designed to increase both awareness and respect for these deeply ingrained cultural practices.

## Data availability statement

The raw data supporting the conclusions of this article will be made available by the authors, without undue reservation.

## Ethics statement

Ethical approval to conduct this study was obtained from the Research Ethics Committee of the University of Sharjah. The studies were conducted in accordance with the local legislation and institutional requirements. The participants provided their written informed consent to participate in this study.

## Author contributions

NA-Y: Formal analysis, Methodology, Supervision, Validation, Writing – original draft, Writing – review & editing. AL: Investigation, Project administration, Writing – original draft. MO: Investigation, Writing – original draft. MA: Investigation, Project administration, Writing – original draft. SI: Project administration, Investigation, Writing – original draft. AH: Project administration, Writing – original draft, Writing – review & editing. FA: Data curation, Methodology, Writing – review & editing. MS: Methodology, Writing – review & editing. JD: Formal analysis, Methodology, Writing – review & editing. SR: Methodology, Validation, Writing – review & editing. AS: Conceptualization, Data curation, Writing – review & editing. HH: Conceptualization, Data curation, Investigation, Writing – review & editing.
